# Acute Lower Limb Ischaemia as a Presenting Sign of Atrial Myxoma: Case Report and Scoping Review of the Literature

**DOI:** 10.1016/j.ejvsvf.2024.07.036

**Published:** 2024-07-08

**Authors:** Alberto M. Settembrini, Leonardo Foresti, Paolo Verlato, Gianluca Buongiovanni, Daniele Bissacco, Chiara Lomazzi, Marco Maggioni, Santi Trimarchi

**Affiliations:** a Department of Cardio Thoracic and Vascular Diseases, Fondazione IRCCS Cà Granda Ospedale Maggiore Policlinico, Milan, Italy; b Department of Vascular Surgery, University Medical Centre Utrecht, Utrecht, the Netherlands; c Division of Pathology, Fondazione IRCCS Ca’ Granda Ospedale Maggiore Policlinico, Milan, Italy; d Clinical and Community Sciences Department, Università degli Studi di Milano, Milan, Italy

**Keywords:** Acute limb ischaemia, Atrial myxoma, Embolectomy, Peripheral embolism

## Abstract

**Objective:**

Cardiac myxomas (CMs) are the most common primary cardiac tumour in adults. They are a rare cause of peripheral embolisation and may present as acute lower limb ischaemia (ALI). A scoping review was undertaken and a case of ALI due to CM embolisation is presented in this paper.

**Methods:**

MEDLINE, Scopus, and Embase were systematically searched for studies reporting data on ALI as a presentation of CM embolisation. The Preferred Reporting Items for Systematic Reviews and Meta-Analyses Extension for Scoping Reviews (PRISMA-ScR) was followed.

**Results:**

A healthy 26 year old female presented to the emergency department with bilateral ALI. Urgent bilateral aorto-iliac embolectomy and distal embolectomy of the left femoropopliteal axis were performed. The retrieved embolic material exhibited a yellowish appearance and jelly like consistency, and histological analysis provided a diagnosis of a myxomatous embolus. Transoesophageal echocardiography confirmed the left atrial origin of a myxomatous tumour, but the residual mass was considered too small for further excision. At a two year clinical follow up, the patient was alive and well without recurrence. Between 1989 and 2023, 59 patients with ALI due to CM embolisation were identified in the literature. An in hospital mortality rate of 12.1% (*n* = 7) was reported, while the in hospital complication and re-intervention rates were 34.5% (*n* = 20) and 27.6% (*n* = 16), respectively. No post-discharge deaths, complications, or re-interventions were reported; fasciotomies were the most reported (*n* = 10). Post-discharge follow up was reported in 22 (37.3%) patients. Mean follow up was 18.0 ± 18.8 months (range 1–120), and 86.4% of patients (*n* = 19) were alive and well at last follow up.

**Conclusion:**

This review and the associated case report underline that CM embolisation should be considered in healthy young patients presenting with cryptogenic ALI. Early transoesophageal echocardiography and histological analysis of the retrieved embolus are recommended to minimise misdiagnosis in these populations.

## Highlights

• Cardiac myxoma embolisation presenting as acute lower limb ischaemia is uncommon.
• Healthy young patients are more likely to be at risk of cardiac myxoma embolisation.
• Transoesophageal echocardiography and histological analysis are suggested.
• Embolectomy combined with cardiac myxomas excision is widely accepted.
• Further studies are needed to assess the role of anticoagulant therapy in recurrences.

## Introduction

Cardiac myxomas (CMs) are the most common type of primary cardiac tumour in adults, originating from the left atrium in 75% of cases, with an estimated annual incidence of 0.5 per million population per year.^1^ Embolic complications occur in 10–45% of CMs, where most myxomatous emboli migrate to the central nervous system or coronary arteries.^2^ However, acute lower limb ischaemia (ALI) may be the initial presentation, and failure to recognise myxomatous emboli can lead to recurrent emboli affecting the prognosis of these patients. This paper reports a case of ALI due to embolisation of CM and a literature review was undertaken to summarise the current knowledge of this scenario. The patient consented to publication of the images.

## Materials and methods

This scoping review adhered to the Preferred Reporting Items for Systematic Reviews and Meta-Analyses Extension for Scoping Reviews (PRISMA-ScR).^3^ The protocol was registered and made publicly accessible on the Open Science Framework (DOI 10.17605/OSF.IO/NHCZB). Two independent investigators systematically reviewed and analysed full text studies published in English within the MEDLINE, Scopus, and Embase databases (last query on 30 August 2023). For discrepancies, a third author was consulted to provide consensus. Research questions and search strings were established prior to the systematic search and are detailed in Supplementary Table S1. The extracted data were analysed using Microsoft Excel and presented in text format, utilising numbers (*n*) and percentages (%), or mean, median ±standard deviation (SD), or range. In accordance with the PRISMA-ScR,^3^ no quality assessment of the included papers was performed.

## Results

### Case report

**Figure 1:**
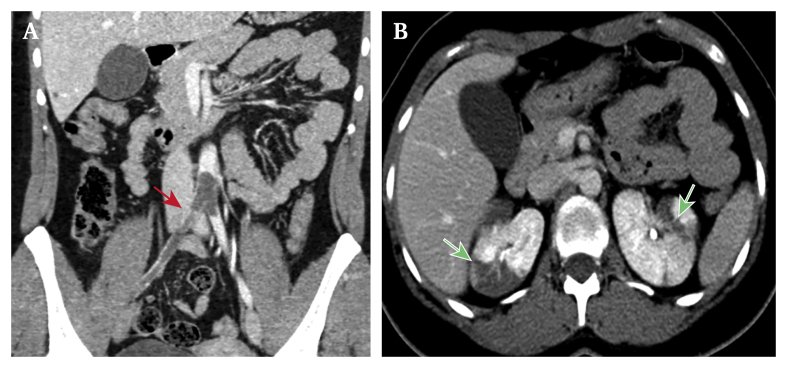
Computed tomography angiography images. (A) Evidence of a saddle embolus of the aortic bifurcation (red arrow) and occlusion of the right iliac axis. (B) Visceral involvement with multiple focal, wedge shaped renal parenchymal defects involving both the cortex and medulla and extending to the capsular surface (green arrows).

A healthy 26 year old Caucasian female presented to the emergency department with bilateral lower limb pain and progressive weakness, with worsening left calf pain, numbness, and cyanotic appearance of the ipsilateral foot after physical exercise. At the first clinical evaluation both femoral pulses were absent, and the patient was diagnosed with ALI, with Rutherford grade^4^ IIa and IIb on the right and left lower limbs, respectively. Transthoracic echocardiography showed no appositions or masses within the cardiac chambers. Duplex ultrasound confirmed the diagnosis, showing absent flow along the left femoropopliteal arterial axis and tardus parvus waveforms in the common femoral arteries, bilaterally. Computed tomography angiography (CTA) showed an occlusion of the aortic bifurcation, with concomitant non-contiguous complete occlusion of the left external iliac and superficial femoral arteries (Fig. 1A). Synchronous splenic and renal infarctions were also observed (Fig. 1B).

**Figure 2:**
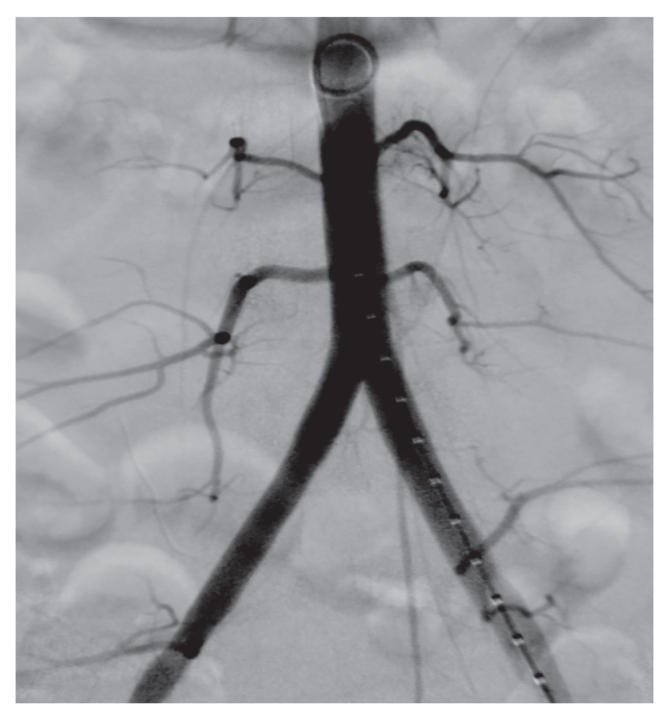
Final angiogram displaying successful aorto-iliac recanalisation after surgical embolectomy.

Under general anaesthesia, the patient underwent urgent bilateral aorto-iliac embolectomy and distal embolectomy of the left femoropopliteal axis using Fogarty catheters. Alongside the recent thrombus, the retrieved embolic material exhibited a yellowish appearance and jelly like consistency, which was submitted to histological analysis. A final angiogram provided confirmation of restoration of the circulation (Fig. 2). Because of significant non-pitting oedema of the left thigh and calf, left limb fasciotomies were performed to prevent compartment syndrome. The patient was transferred to the intensive care unit (ICU) for haemodialysis due to the development of acute renal failure.

**Figure 3:**
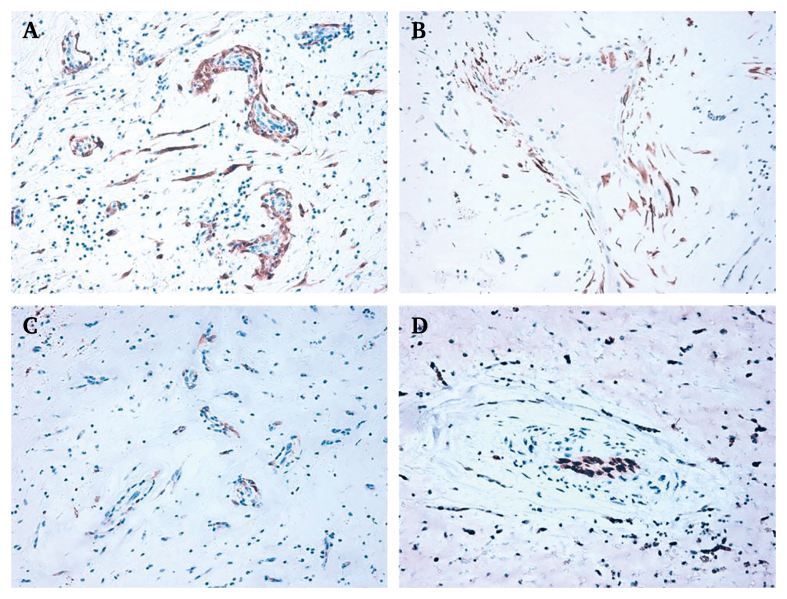
Tumour cell immunoreactivity and identification in the histological analysis of the embolic material. (A) Diffuse immunoreactivity for calretinin and (B) cytokeratin AE1/AE3; (C) focal immunoreactivity for S100 protein; (D) ETS related gene (ERG) highlighting endothelial cells but not perivascular tumour cells (H&E 200x).

Histological analysis confirmed the diagnosis of a myxomatous embolus (Fig. 3). During the ICU stay, transoesophageal echocardiography (TEE) and CTA confirmed the left atrial origin of a myxomatous tumour and ruled out brain infarctions. The residual mass (12 x 7 x 6 mm) was considered too small to require further surgery. Although, all the patient's dorsalis pedis and posterior tibial pulses were restored, the left foot remained slightly dropped with paraesthesia. On post-operative day 6, the patient was discharged from the ICU. During the post-operative course, the patient showed complete clinical regression of the deficits and a progressive improvement of renal function, which led to hospital discharge on post-operative day 41. Short term prophylactic anticoagulation (low molecular weight heparin4 000 UI/day for 14 days) was continued beyond discharge. The follow up consisted of clinical and Duplex ultrasound evaluations at six and 12 months, and yearly thereafter. At the two year follow up, the patient was alive and asymptomatic.

### Scoping review

The systematic search identified 553 studies. Fifty one studies were included after the selection process (Supplementary Fig. S1),^5^, ^6^, ^7^, ^8^, ^9^, ^10^, ^11^, ^12^, ^13^, ^14^, ^15^, ^16^, ^17^, ^18^, ^19^, ^20^, ^21^, ^22^, ^23^, ^24^, ^25^, ^26^, ^27^, ^28^, ^29^, ^30^, ^31^, ^32^, ^33^, ^34^, ^35^, ^36^, ^37^, ^38^, ^39^, ^40^, ^41^, ^42^, ^43^, ^44^, ^45^, ^46^, ^47^, ^48^, ^49^, ^50^, ^51^, ^52^, ^53^, ^54^, ^55^ including 59 patients. Study details are listed in Supplementary Table S2.

The median age at presentation was 44.5 years (range 0.5–82), with half of the patients being female. Rutherford's grade IIb ALI was the most observed at presentation (*n* = 21), and systemic embolisation was noted in 31 patients. The most common site of embolisation leading to ALI was the infrarenal aorta, including the aortic bifurcation (*n* = 26). Clinical examination combined with CTA was the most frequently used diagnostic method. Details regarding demographics and diagnosis are provided in Supplementary Table S3. The mean maximum diameter of the CM, mostly located in the left atrium, was 4.2 ± 2.1 mm. Details of the medical and surgical management are listed in Table 1.

**Table 1:** Medical and surgical management strategies for acute lower limb ischaemia following cardiac myxoma embolisation described in the literature.

Medical and surgical management	*n* (%)
*Anticoagulation regimen*	13 (22.0)
Pre-operative	8 (61.5)
Pre-operative + post-operative	2 (15.4)
None	3 (23.1)
*Surgical strategy*	
Embolectomy	55 (93.2)
Peripheral bypass	2 (3.4)
Above knee amputation	1 (1.7)
Not reported	1 (1.7)
*Cardiac myxoma location*	
Left atrium	47 (79.7)
Multichambered	1 (1.7)
Not reported	11 (18.6)
*Cardiac myxoma diagnosis method*	
TTE or TEE + biopsy	27 (45.8)
TTE or TEE	10 (16.9)
Embolus histological analysis	2 (3.4)
CTA + TEE	4 (6.8)
Not reported	16 (27.1)
Cardia myxoma dimensions – mm ± SD	4.2 ± 2.1
*Cardiac myxoma excision*	
Surgical excision	39 (66.1)
No excision of the residual mass	7 (11.9)
Complete embolisation	8 (13.6)
Not reported	5 (8.4)

CTA = computed tomography angiography; TEE = transoesophageal echocardiography; TTE = transthoracic echocardiography; CTA = computed tomography angiography.

Fifty of the 51 studies reported data on in hospital outcomes, with an overall mortality rate of 12.1% (*n* = 7). The observed in hospital complication rate was 34.5% (*n* = 20), while in hospital re-interventions were observed in 27.6% of patients (*n* = 16). Details about post-discharge follow up were noted for 22 patients, with a mean follow up of 18.0 ± 18.8 months (range 1–120). No deaths, complications, or re-interventions were reported in this group. Follow up details about outcomes are listed in Table 2.

**Table 2:** Outcomes of patients with acute lower limb ischaemia following cardiac myxoma embolisation.

Outcomes	*n* (%)
*Death*	7 (12.1)
In hospital	6 (85.7)
Post-discharge∗	0 (0)
Not reported	1 (14.3)
*Cause of death*	
Not reported	4 (57.1)
Stroke	2 (28.6)
Neurological deterioration	1 (14.3)
*Complications*	
*In hospital*	20 (34.5)
Stroke	5 (8.6)
ARF requiring haemodialysis	5 (8.6)
Compartment syndrome	3 (5.2)
Pulmonary	2 (3.4)
CLTI	2 (3.4)
ALI	1 (1.7)
Lower limb paralysis	1 (1.7)
ARF not requiring haemodialysis	1 (1.7)
*Post-discharge*∗	0 (0)
*Re-interventions*	
*In hospital*	16 (27.6)
Fasciotomies	10 (17.2)
Lower limb amputation	3 (5.2)
Decompressive craniotomy	2 (3.4)
New embolectomy	1 (1.7)
*Post-discharge*∗	0 (0)
Mean hospitalisation – days ±SD	15.6 ± 12.0
Patient with post-discharge follow up	22 (37.3)
Follow up – months ±SD [range]	18.0 ± 18.8 [1–120]
*Clinical condition of the patient at last follow up*	
Alive and well	19 (86.4)
Alive with limitations in everyday life	2 (9.1)
Alive with paralysis	1 (4.5)

ALI = acute lower limb ischaemia; ARF = acute renal failure; CLTI = chronic limb threatening ischaemia; SD = standard deviation.

∗ Post-discharge data only refer to patients with post-discharge follow up data (*n* = 22).

## Discussion

This case report adds to the other 59 cases reported in the literature describing acute lower limb ischaemia as a presentation of CM embolisation. The patient, a young female with no prior medical history, aligned with the typical demographic characteristics observed in the literature. As expected, the preferred surgical management strategy for such cases involves embolectomy combined with a second stage CM excision, when feasible. The decision to make a CM excision was primarily driven by the risk of recurrent CM embolisation, which could affect the vital organs. As shown in this review, brain embolisation or involvement of renal and splanchnic arteries are not uncommon and appear to be significant factors influencing outcomes in this population. These findings underscore the importance of early identification of the embolus origin. For these reasons, particularly in young patients with cryptogenic ALI, it is suggested that additional vascular beds be examined at the time of diagnosis and a pre-operative echocardiogram, preferably TEE when feasible, be performed.^11^

However, as shown in this case, diagnosing CM embolisation can be challenging, especially when there is complete or near complete detachment of the tumour at the time of embolisation, which could evade diagnostic imaging.^17^ Although this event may reduce or eliminate the risk of recurrent emboli, performing a histological examination of the surgically retrieved embolic material is suggested in cases of cryptogenic ALI to avoid a missed diagnosis.

One of the major concerns about the management of CM embolisation is the management of anticoagulation therapy (ACT), and this review showed a lack of data regarding this topic. Some authors suggested ACT even after complete detachment of the tumour, based on residual endothelial abnormalities that may lead to recurrence even after several years.^17^ Otherwise, long term post-operative ACT has been proven ineffective in preventing further recurrent embolic episodes,^1^ and its therapeutic value remains uncertain, especially after primary tumour excision or complete detachment. Based on this heterogeneity and the lack of consensus in the literature, assessing the risk of recurrence and tailoring treatment to the patient are suggested with prolonged ACT. Further studies are needed to establish the best medical management in these patients.

A recent meta-analysis reported an adjusted estimate rate of CM recurrence after surgical excision of 0.03 cases per one 1 000 person years.^56^ The current case had near complete embolisation of the tumour, leaving a residual mass that was difficult to characterise in size. Along with cardiac surgeons, the team considered this to be a radical excision and decided not to perform a high risk and highly invasive procedure due to the low risk of recurrence. However, while there are no reported cases of recurrence following complete CM embolisation, long term surveillance through repeated imaging may still be indicated for vigilant monitoring. This is particularly important for patients with CMs that have multi-chamber distribution, early distant metastases, atypical origin, and family history.

### Conclusion

Cardiac myxoma embolisation presenting as acute lower limb ischaemia is uncommon, with a total of 60 cases reported in the literature, including this one. However, CM embolisation should be considered in healthy young patients presenting with cryptogenic ALI, especially if combined with systemic embolisation, and an early TEE and histological analysis of the retrieved embolus should be considered in these populations. While surgical management with embolectomy combined with CM excision is widely accepted, further studies are needed to assess the importance of ACT in preventing recurrence.

## Conflict of interest

The authors declare that they have no known competing financial interests or personal relationships that could have appeared to influence the work reported in this paper.

## Acknowledgements

This study was partially funded by Italian Ministry of Health, current research IRCCS.

## Reporting checklist

The authors have completed the CARE reporting checklist. Available at https://cdt.amegroups.com/article/view/10.21037/cdt-22-401/rc.

## Ethical statement

The authors are accountable for all aspects of this work, in ensuring that questions related to the accuracy or integrity of any part of the work were appropriately investigated and resolved. All procedures performed in this study were in accordance with the ethical standards of the institutional and or national research committee(s) and with the Helsinki Declaration (as revised in 2013). Written informed consent was obtained from the patient for publication of this case report and accompanying images. A copy of the written consent is available for review by the editorial office of this journal.
