# Supplementary material for: Acute Lower Limb Ischaemia as a Presenting Sign of Atrial Myxoma: Case Report and Scoping Review of the Literature
Source: EJVES Vasc Forum. 2024 Jul 8;62:35–40. doi: 10.1016/j.ejvsvf.2024.07.036 (PMC11422033; doi:10.1016/j.ejvsvf.2024.07.036)

**Supplementary Table S1.** Research question and search strings.

| **Research question** | ‘What are the current management strategies and the associated outcomes for acute lower limb ischemia due to cardiac myxoma embolization?’ |
| --- | --- |
| **Search strings** | MEDLINE: ("Heart Neoplasms"[Mesh] OR "atrial myxoma") AND ("Arterial Occlusive Diseases"[Mesh] OR "peripheral ischemia" OR "limb ischemia"))  EMBASE: ('heart myxoma'/exp OR 'heart atrium myxoma'/exp OR 'atrial myxoma' OR 'atrium myxoma' OR 'cardiac atrial myxoma' OR 'cardiac atrium myxoma' OR 'heart atrial myxoma' OR 'heart atrium myxoma' OR 'myxoma in the right atrium' OR 'myxoma, heart atrium' OR 'myxoma, right atrium' OR 'right atrium myxoma' OR 'right heart atrium myxoma') AND ('ischemia'/exp OR 'blood circulation disorder' OR 'blood flow disorder' OR 'circulation disorder' OR 'circulation failure' OR 'circulatory disorder' OR 'circulatory disturbance' OR 'circulatory failure' OR 'ischaemia' OR 'ischaemic disease' OR 'ischaemic episode' OR 'ischaemic event' OR 'ischaemic syndrome' OR 'ischemia' OR 'ischemic disease' OR 'ischemic episode' OR 'ischemic event' OR 'ischemic syndrome' OR 'tissue ischaemia' OR 'tissue ischemia' OR 'warm ischaemia' OR 'warm ischemia' OR 'thromboembolism'/exp OR 'cerebral embolism and thrombosis' OR 'embolism and thrombosis' OR 'embolism, thrombo' OR 'intracranial embolism and thrombosis' OR 'thrombo embolic disease' OR 'thrombo embolism' OR 'thrombo-emboli' OR 'thrombo-embolus' OR 'thromboemboli' OR 'thromboembolic' OR 'thromboembolic complication' OR 'thromboembolic disease' OR 'thromboembolic process' OR 'thromboembolism' OR 'thromboembolus' OR 'thromboemboly') AND 'limb'/exp  SCOPUS: ALL ( ( "heart myxoma" OR "atrium myxoma" OR "atrial myxoma" OR "myxoma in the left atrium" OR "left atrium myxoma" OR "left atrial myxoma" ) AND ( "ischemia" OR "ischaemia" OR "ischaemic disease" OR "ischaemic" OR "thromboembolism" OR "thrombo embolic disease" OR "thrombo" OR "embolism" OR "thrombo-emboli" OR "thrombo-embolus" OR "thromboemboli" OR "thromboembolic" OR "thromboembolic complication" OR "thromboembolic disease" OR "thromboembolism" OR "thromboembolus" OR "thromboemboly" ) AND ( "limb" ) ) |

**Supplementary Table S2.** Characteristics of the included studies.

|  | **Author, year** | **Study design** | **Study period** | **Country** | **No. of patients** |
| --- | --- | --- | --- | --- | --- |
| **1** | Abdul Uloom, 2013 | Case Report | - | Auckland, New Zealand | 1 |
| **2** | Abdull Gaffar, 2018 | Retrospective, observational, single-centre study | 2005 - 2017 | Dubai, United Arab Emirates | 3 |
|  |  |  |  |  |  |
|  |  |  |  |  |  |
| **3** | Ahmed, 2007 | Letter to editor | - | UK | 1 |
| **4** | Ali, 2004 | Case Report | - | UK | 1 |
| **5** | Ammar, 2007 | Case Report | - | UK | 1 |
| **6** | Bernatchez, 2018 | Case Report | - | Canada | 1 |
| **7** | Bois, 2018 | Retrospective, observational, single-centre study | Jan 1998 - Dec 2014 | USA | 2 |
| **8** | Cengiz Çolak, 2011 | Case Report | - | Turkey | 1 |
| **9** | Eriksen, 1992 | Case Report | - | Denmark | 1 |
| **10** | Ermacora, 2022 | Case Report | - | Italy | 1 |
| **11** | Fang, 2003 | Case Report | - | China | 1 |
| **12** | Habbab, 2014 | Case Report | - | Canada | 1 |
| **13** | Hiramoto, 2005 | Case Report | - | USA | 1 |
| **14** | Ho, 2020 | Case Report | - | Australia | 1 |
| **15** | Hofer, 2009 | Case Report | - | Austria | 1 |
| **16** | Horn, 1997 | Case Report | - | USA | 1 |
| **18** | Kapur, 2006 | Case Report | - | USA | 1 |
| **19** | Kaul, 2012 | Case Report | - | UK | 1 |
| **20** | Kawabata, 2015 | Case Report | - | Japan | 1 |
| **21** | Keshelava, 2018 | Case Report | - | USA | 1 |
| **22** | Knight, 2015 | Case Report | - | South Africa | 1 |
| **23** | Kumar, 2023 | Case Report | - | India | 1 |
| **24** | Latifi, 2019 | Case Report | - | USA | 1 |
| **25** | Lee, 2012 | Retrospective, observational, single-centre study | Jan 2000 - Mar 2011 | Republic of Korea | 1 |
| **26** | Li, 2021 | Case Report | - | Germany | 1 |
|  |  |  |  |  |  |
| **28** | Mathew, 2019 | Case Report | - | India | 1 |
| **29** | McMullin, 1993 | Case Report | - | Australia | 1 |
| **30** | Meng, 2022 | Case Report | - | Japan | 1 |
| **31** | Min, 2014 | Case Report | - | South Korea | 1 |
| **32** | Miroslav, 2005 | Retrospective, observational, single-centre study | 1983-2003 | Serbia and Montenegro | 2 |
| **33** | Mohamed, 2018 | Case Report | - | USA | 1 |
| **34** | Neff, 2008 | Case Report | - | USA | 1 |
| **35** | Nicholls, 2012 | Case Report | - | New Zealand | 1 |
| **36** | Salimi, 2015 | Case Report | - | Iran | 1 |
| **37** | Shavit, 2007 | Case Report | - | Israel | 1 |
| **38** | Zhou, 2023 | Case Report | - | China | 1 |
| **39** | Szymanska, 2019 | Image | - | Poland | 1 |
| **40** | Taşdemir, 2000 | Case series | 1991-1998 | Turkey | 2 |
| **41** | Tasoglu, 2009 | Retrospective, observational, single-centre study | 1990-2006 | Turkey | 3 |
| **42** | Tsao, 2010 | Image | - | Taiwan | 1 |
| **43** | Val-Bernal, 2003 | Case Series | 1973–2000 | Spain | 2 |
| **44** | Van der Mieren, 2016 | Case Report | - | Belgium | 1 |
| **45** | Weerasena, 1989 | Case Report | - | UK | 1 |
| **46** | Wilson, 1997 | Case Report | - | UK | 1 |
| **47** | Wu, 2018 | Case Report | - | China | 1 |
| **48** | Yadav, 2009 | Case Report | - | Australia | 1 |
| **49** | Yamashita, 2018 | Case Report | - | Japan | 1 |
| **50** | Zhang J, 2006 | Case Report | - | China | 1 |
| **51** | Zhang T, 2012 | Case Report | - | China | 1 |
| **52** | Zuin, 2017 | Image Focus | - | Italy | 1 |
| **53** | Zulfa, 2023 | Case Report | - | Indonesia | 1 |

**Supplementary Table S3.** Demographics and diagnosis of patients with acute lower limb ischaemia due to cardiac myxoma embolisation.

| **Author, year** | **Age,**  **mean ± SD [range], years** | **Sex, n** | **Rutherford classification, laterality, n** | **Imaging** | **Site of embolization causing ischemia, n** | **Arterial embolization territory, n** |
| --- | --- | --- | --- | --- | --- | --- |
| Abdul Uloom,  2013 | 21 | F, 1 | Not determinable due to neurological involvement. | CTA | AAA, 1  Iliac axis, bilateral, 1  Infrapopliteal, unilateral, 1 | Cerebrovascular, 1  Renal, 1  Splenic, 1 |
| Abdull Gaffar,  2018 | 49.3 ± 8.7  [38-59] | F, 2;  M, 1 | IIb, 3 (100), bilateral 1 (33.3), unilateral 2 (66.7) | - | Iliac axis, bilateral, 1  Femoral axis, unilateral, 1  Infrapopliteal, unilateral, 1 | - |
| Ahmed,  2007 | 45 | F, 1 | IIa, unilateral | CTA | Iliac axis, unilateral, 1  Femoral axis, unilateral, 1 | - |
| Ali,  2004 | 58 | M, 1 | IIb, bilateral | - | AAA, 1 | - |
| Ammar,  2007 | 10 | M, 1 | IIb, bilateral | CTA | Aortic bifurcation, 1 | - |
| Bernatchez  2018 | 45 | M, 1 | IIb, bilateral | CTA | Aortic bifurcation, 1  Iliac axis, bilateral, 1  Femoral axis, bilateral, 1 | Splanchnic, 1 |
| Bois,  2018 | 48.5  [42-55] | M, 2 | NR | - | AAA, 1  Femoral axis, bilateral, 1  Infrapopliteal, bilateral, 1 | - |
| Cengiz Çolak,  2011 | 46 | M, 1 | IIb, bilateral | - | Iliac axis, bilateral, 1,  Femoral axis, bilateral, 1 | Cerebral, 1  Splanchnic, 1  Renal, 1 |
| Eriksen,  1992 | 41 | F, 1 | Not determinable due to neurological involvement. | Angiography | Aortic bifurcation, 1 | Cerebral, 1 |
| Ermacora,  2022 | 52 | F, 1 | IIa, bilateral | CTA | Iliac axis, bilateral, 1  Infrapopliteal, unilateral, 1 | Cerebral, 1  Splanchnic, 1  Splenic, 1  Renal, 1 |
| Fang,  2003 | 53 | M, 1 | IIb, bilateral | Angiography | AAA, 1 | Renal, 1 |
| Habbab,  2014 | 52 | F, 1 | Unconscious | CTA | AAA, 1  Iliac axis, bilateral, 1  Femoral axis, bilateral, 1  Infrapopliteal, unilateral, 1 | Cerebral, 1  Splenic, 1  Renal, 1 |
| Hiramoto,2005 | 39 | M, 1 | IIb, unilateral (left) | Angiography | Femoral axis, unilateral, 1 | - |
| Ho,  2020 | 50 | F, 1 | IIa, unilateral (right) | CTA | Infrapopliteal, bilateral, 1 | Renal, 1 |
| Hofer,  2009 | 51 | F, 1 | III, bilateral | CTA | AAA, 1 | Renal, 1 |
| Horn,  1997 | 42 | F, 1 | IIb, bilateral | DUS+ Angiography | AAA, 1  Iliac axis, bilateral, 1 | - |
| Kapur,  2006 | 30 | M, 1 | I, bilateral | DSA | AAA, 1 | - |
| Kaul,  2012 | 56 | M, 1 | IIb, bilateral | CTA | AAA, 1  Iliac axis, bilateral, 1 | - |
| Kawabata,  2015 | 0.5 | F, 1 | NR | CTA | Suprarenal aorta,  Femoral axis, bilateral, 1 | Splanchnic, 1 |
| Keshelava,  2018 | 37 | M, 1 | Not determinable due to neurological involvement. | CTA | AAA, 1 | Renal, 1  Spinal cord, 1 |
| Knight,  2015 | 41 | M, 1 | NR | CTA | - | Cerebral, 1 |
| Kumar,  2023 | 34 | F, 1 | IIa, bilateral | CTA | AAA, 1  Iliac axis, bilateral, 1 | Upper limb, 1 |
| Latifi,  2019 | 61 | F, 1 | IIa, bilateral | DUS+CTA | Iliac axis, unilateral, 1  Femoral axis, unilateral, 1 | Splenic, 1  Renal, 1 |
| Lee,  2012 | 55 | F, 1 | NR | - | Iliac axis, unilateral, 1 | - |
| Li,  2021 | 21 | F, 1 | NR | - | Aortic bifurcation, 1  Iliac axis, bilateral, 1  Femoral axis, bilateral, 1 | Cerebral, 1  Splenic, 1  Renal, 1 |
| Mathew,  2019 | 18 | F, 1 | IIb, bilateral | DUS | AAA, 1  Iliac axis, unilateral, 1  Femoral axis, unilateral, 1 | - |
| McMullin,  1993 | 50 | M, 1 | IIb, bilateral | - | Aortic bifurcation, 1 | - |
| Meng,  2022 | 67 | M, 1 | IIb, unilateral (left) | CTA | Infrapopliteal, unilateral, 1 | - |
| Min,  2014 | 36 | F, 1 | Not determinable due to neurological involvement. | CTA | Aortic bifurcation, 1  Iliac axis, bilateral, 1  Infrapopliteal, bilateral, 1 | Cerebral, 1  Splenic, 1  Renal, 1 |
| Miroslav,  2005 | 38.5  [35-42] | M, 1;  F, 1 | NR | - | Femoral axis, unilateral, 1  Infrapopliteal, unilateral, 1 | - |
| Mohamed,  2018 | 55 | M, 1 | IIa, unilateral (right) | CTA | Femoral axis, unilateral, 1  Infrapopliteal, unilateral, 1 | - |
| Neff,  2008 | 45 | F, 1 | Not determinable due to neurological involvement. | CTA | AAA, 1  Iliac axis, bilateral, 1 | Cerebral, 1 |
| Nicholls,  2012 | 44 | M, 1 | Not determinable due to neurological involvement. | CTA | Iliac axis, unilateral, 1 | Cerebral, 1  Splenic, 1  Renal, 1 |
| Salimi,  2015 | 78 | M, 1 | IIb, unilateral (left) | DUS+CTA | Femoral axis, unilateral, 1  Infrapopliteal, unilateral, 1 | Cerebral, 1 |
| Shavit,  2007 | 42 | M, 1 | NR | CTA | AAA, 1  Iliac axis, bilateral, 1 | Splenic, 1  Renal, 1 |
| Zhou,  2023 | 81 | F, 1 | IIb, unilateral (right) | DUS+CTA | Femoral axis, unilateral, 1 | - |
| Szymanska  2019 | 82 | F, 1 | IIb, unilateral (left) | DUS | Femoral axis, unilateral, 1 | - |
| Taşdemir,  2000 | 23  [20- 26] | F, 1;  M, 1 | NR | - | - | Upper limb, 1 |
| Tasoglu,  2009 | - | - | NR | - | - | - |
| Tsao,  2010 | 55 | M, 1 | IIb, bilateral | CTA | Descending thoracic aorta | Splanchnic, 1  Renal, 1 |
| Val-Bernal,  2003 | 46  [55-37] | F, 2 | IIb, unilateral (left), 2 | - | Femoral axis, unilateral, 2 | Upper limb, 1 |
| Van der Mieren,  2016 | 50 | M, 1 | IIb, bilateral | CTA | AAA, 1  Iliac axis, bilateral, 1  Femoral axis, unilateral, 1 | Splanchnic, 1  Splenic, 1  Renal, 1 |
| Weerasena,  1989 | 14 | F, 1 | Not determinable due to neurological involvement. | - | Aortic bifurcation, 1 | - |
| Wilson,  1997 | 43 | F, 1 | IIa, unilateral (left) | Angiography | Femoral axis, unilateral, 1  Infrapopliteal, unilateral, 1 | - |
| Wu,  2018 | 16 | M, 1 | Not determinable due to neurological involvement. | CTA | Infrapopliteal, bilateral, 1 | Cerebral, 1 |
| Yadav,  2009 | 62 | F, 1 | Not determinable due to neurological involvement. | CTA | AAA, 1  Iliac axis, bilateral, 1 | Upper limb, 1  Pulmonary, 1  Splenic, 1  Renal, 1 |
| Yamashita,  2018 | 45 | M, 1 | IIa, unilateral (right) | CTA | Femoral axis, unilateral, 1 | Cerebral, 1  Splenic, 1 |
| Zhang J,  2006 | 35 | M, 1 | IIb, bilateral | DUS | AAA, 1  Iliac axis, bilateral, 1 | Renal, 1 |
| Zhang T,  2012 | 29 | M, 1 | IIa, bilateral | CTA | AAA, 1  Iliac axis, bilateral, 1  Femoral axis, bilateral, 1 | Splenic, 1  Renal, 1 |
| Zuin,  2017 | 63 | M, 1 | IIa, bilateral | CTA | AAA, 1  Iliac axis, bilateral, 1 | Splenic, 1  Renal, 1 |
| Zulfa,  2023 | 23 | F, 1 | NR | CTA | - | Cerebral, 1 |

AAA = Infrarenal aorta; CIA = Common Iliac Artery; CTA = Computed Tomography Angiography; DUS = Duplex Ultrasound; PA = Popliteal Artery.

**Supplementary Figure S1.** PRISMA flow chart.


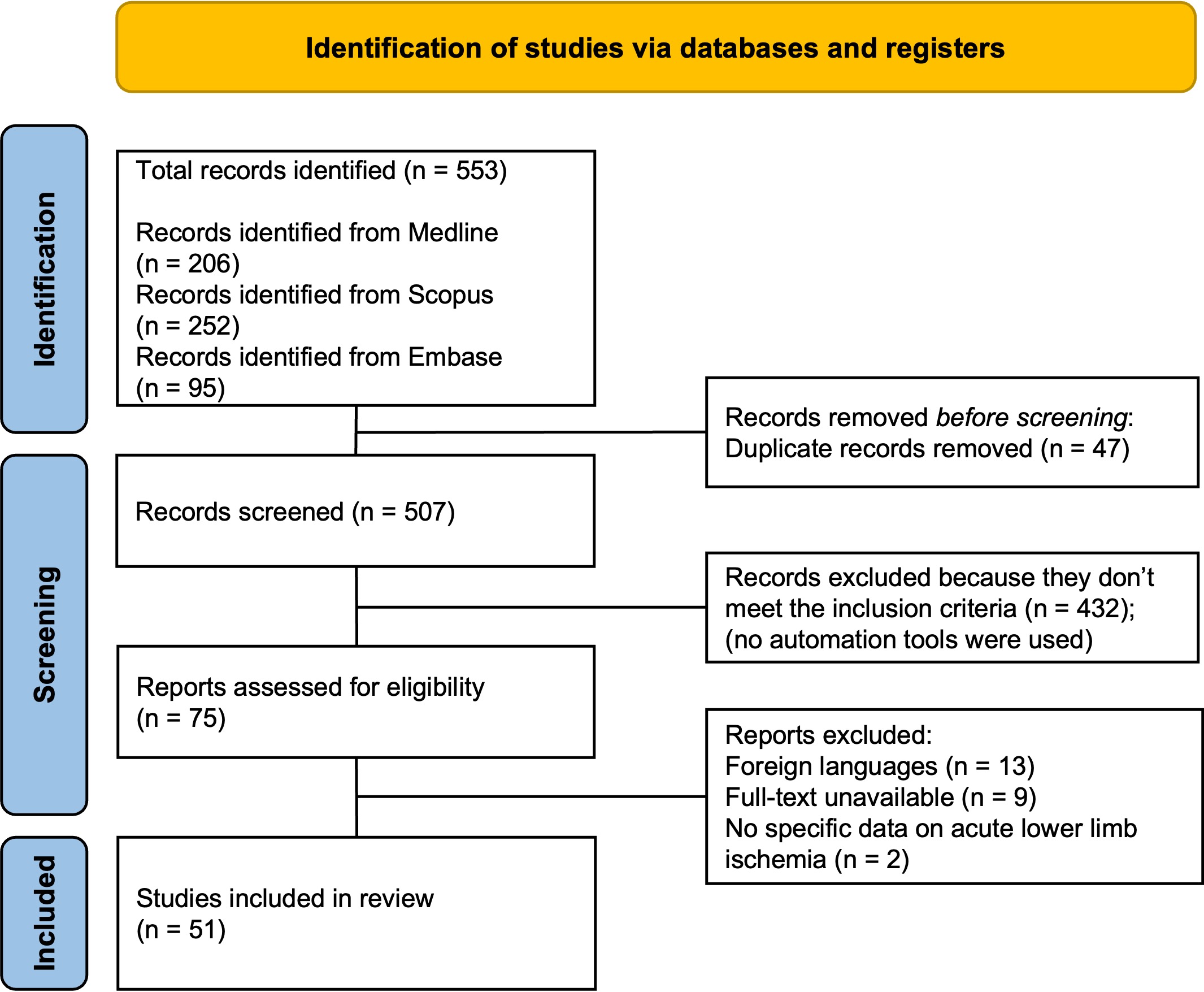

Supplement: Multimedia component 1 [file mmc1.docx]
